# Archaeobotanical Study of Ancient Food and Cereal Remains at the Astana Cemeteries, Xinjiang, China

**DOI:** 10.1371/journal.pone.0045137

**Published:** 2012-09-20

**Authors:** Tao Chen, Yan Wu, Yongbing Zhang, Bo Wang, Yaowu Hu, Changsui Wang, Hongen Jiang

**Affiliations:** 1 The Lab of Human Evolution, Institute of Vertebrate Paleontology and Paleoanthropology, Chinese Academy of Sciences, Beijing, China; 2 Department of Scientific History and Archaeometry, Graduate University of Chinese Academy of Sciences, Beijing, China; 3 Academia Turfanica of Xinjiang Uygur Autonomous Region, Turpan, China; 4 Xinjiang Uygur Autonomous Region Museum, Urumchi, China; KULeuven, Belgium

## Abstract

Starch grain, phytolith and cereal bran fragments were analyzed in order to identify the food remains including cakes, dumplings, as well as porridge unearthed at the Astana Cemeteries in Turpan of Xinjiang, China. The results suggest that the cakes were made from *Triticum aestivum* while the dumplings were made from *Triticum aestivum*, along with *Setaria italica*. The ingredients of the porridge remains emanated from *Panicum miliaceum*. Moreover, direct macrobotantical evidence of the utilization of six cereal crops, such as *Triticum aestivum*, *Hordeum vulgare* var. *coeleste*, *Panicum miliaceum*, *Setaria italica*, *Cannabis sativa*, and *Oryza sativa* in the Turpan region during the Jin and Tang dynasties (about 3^rd^ to 9^th^ centuries) is also presented. All of these cereal crops not only provided food for the survival of the indigenous people, but also spiced up their daily life.

## Introduction

Revealing what our ancestors ingested has been and is still such a fascinating quest for both archaeologists and the general public. In most cases, animal remains, plant macrofossils, and food residues are often regular materials for palaeodietary investigation [1]–[3]. In addition, stable isotope and trace element analysis of human bone can also enhance our understanding of ancient foods [4], [5]. Unlike other materials, the information gleaned by food remains is far more straightforward. Nevertheless, due to poor conditions of preservation, food remains are rarely encountered in archaeological excavations. The Turpan Basin of Xinjiang, China, is characterized by a dry climate, which has favored the preservation and prevented the decay of numerous mummies as well as plant and food remains. For example, a number of significant plant remains, *e.g.*, *Triticum aestivum*, *Hordeum vulgare* var. *coeleste*, *Panicum miliaceum*, *Cannabis sativa*, *Lithospermum officinale*, *Capparis spinosa*, *Vitis vinifera*, etc., have been well studied over the past several years [6]–[10]. Processed food like noodles and cakes have been occasionally discovered at archaeological sites, such as the Subeixi Site [11], and the Sampula and Yingpan Cemeteries [12], [13]. This kind of physical evidence provides opportunities to trace the vegetation patterns, palaeodiet and plant utilization among the ancient indigenous people of Xinjiang. However, aside from the ancient noodles and cakes unearthed at the Subeixi site [14], to date, the overwhelming majority of food remains have not been scientifically analyzed.

As is known by many, the human diet experienced a change: from a simpler to more complicated one; from a more primitive to a more developed one. In view of different natural environments and cultural backgrounds, people who lived in different places and ages had different characteristics of dietary culture. Turpan is located in the eastern part of Central Asia, an important communication center between the East and the West. Due to its unique natural geographical environment, its location on important routes of exchange, and patterns of historical development, the indigenous people in Turpan have had their own unique food culture since ancient times. Previous studies have revealed only part of the cereals processed during the period 1000-100BC in Turpan [14]. However, the diet which characterized this region during the later times has not yet well been investigated. The primary objective of the present study is to reveal the burial ritual, dietary and food culture of the people of ancient Turpan during the period 200–900AD. Concurrently, the cultural information exchanged underlying these food and cereal crops remains are further explored.

### Site description

**Figure 1:**
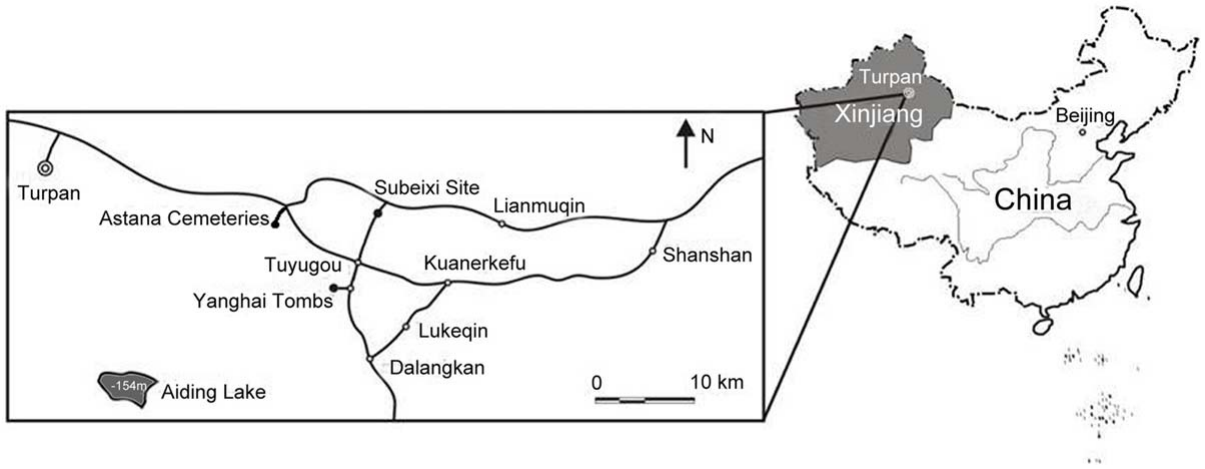
Map of the location of the Astana Cemeteries (adapted from [**14**], modified).

Previous studies have shown that the Turpan Basin was occupied by the Gushi people from 1000 to 100BC. The Gushi Kingdom was later defeated by the armies of the Western Han dynasty (202 BC-9 AD), and large numbers of Han Chinese migrated to the region. Afterwards, Turpan was successively dominated by kingdoms consisting of the Han people, especially the Gaochang Kingdom, until the 9^th^ century [15]. The Astana Cemeteries were the public graveyards of the ancient Gaochang people, covering an area of 10 km^2^. The cemeteries are located in front of the Flaming Mountains (Huoyan Shan) and lie on the delta plain of the Mutougou River (Fig. 1). They consist of thousands of tombs dating from the 3^rd^ to the 9^th^ centuries, and are well known today as an “underground museum”. As Turpan is famous for its hot and dry climate, as previously published [6]–[10], the mummies, together with the funerary objects, have been well preserved without decay. From 1959 to 1975, more than 500 tombs were excavated by the Xinjiang Uyghur Autonomous Region Museum and other institutions. According to the structure and unearthed cultural relics, all the tombs can be divided into three chronological phases. The first phase existed between the Jin Dynasty and middle of the Southern and Northern dynasties (middle 3^rd^ to early 6^th^ centuries AD). According to their shape, the tombs belonging to this period can be categorized into two different types: the first is a cave-cum-shaft grave with a slope, most of which are square, with four straight walls, while some have side chambers; The second is a cave-cum-shaft grave with a silo. The second phase dates from between the middle of the Southern and Northern dynasties and the early Tang dynasty (early 6^th^ to middle 7^th^ centuries AD), namely the time of Gaochang Kingdom. During this period, the walls of tombs became slightly curved, with flat and round tops. Wooden coffins were no longer used. The last phase dates to the early-middle Tang dynasty (middle 7^th^ to the 9^th^ centuries AD). During this period, the surfaces of tombs gradually evolved from flat to circular. Additionally, the dual-chamber tomb began to appear [16].

## Materials and Methods

### Ethics Statement

All necessary permits were obtained from the Academia Turfanica of Xinjiang Uygur Autonomous Region and Xinjiang Uygur Autonomous Region Museum for the described field studies.

**Figure 2:**
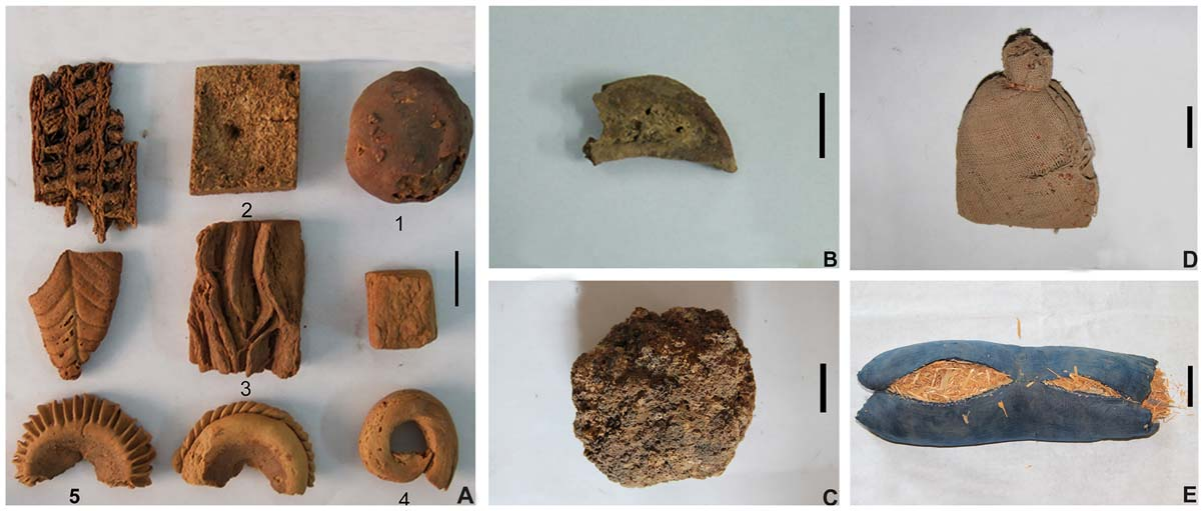
Materials chosen for microfossil analysis. (A) Five cakes labeled 1–5 chosen for microfossil analysis. Scale bar = 2 cm; (B) Dumping chosen for microfossil analysis. Scale bar = 2 cm; (C) Desiccated porridge chosen for microfossil analysis. Scale bar = 2 cm; (D) A small bag of *Setaria italica* from tomb No. 64TAM5:37. Scale bar = 2 cm; (E) A pillow from tomb No. 73TAM519:18 using stalks of *Triticum aestivum* and *H. vulgare* var. *coeleste* as filling. Scale bar = 5 cm.

In the past, plant materials were an important constituent of funerary objects. In fact, some cereals were carefully packaged or stored in earthen pots or small bags (Fig. 2D), and buried in the tombs for further use by the deceased; some plant materials were discovered in objects of daily use, *e.g.*, stalks of *Triticum aestivum* used for the filling of pillows (Fig. 2E). In view of the very large number of tombs, we adopted the method of random sampling. Judging from the structure of tombs and unearthed cultural relics, the deceased were mainly from high-status part of the ancient society of Turpan. Samples were chosen for micro-botanical analyses, including cakes of different shapes from tomb No. 64TAM37:11, porridge from tomb No. 64TAM13:15, as well as dumplings from tomb No. 73TAM514:5 (Fig. 2 and Table 1). Some cereal remains unearthed from some tombs in the Astana Cemeteries were also identified (Table 1). The specimens were subsequently deposited in the Xinjiang Uyghur Autonomous Region Museum as were modern reference collection samples deposited in the Department of Scientific History and Archaeometry, Graduate University of Chinese Academy of Sciences.

**Table 1:** Data regarding unearthed cereal and food remains from tombs.

Tomb number	Identified cereal taxa or food remain	Storage condition	Age	Origin of age	Quantity
04TAM409:10	*Panicum miliaceum*, *Oryza sativa*	Grain with husk	304–439 AD	Structure of tomb and unearthed cultural relics	*P. miliaceum* mixed with small amout of *O. sativa* stored in a pot basin
73TAM514:5	Dumpling made from *T. aestivum and S. italica*		499–640AD	Structure of tomb and unearthed cultural relics	2
60TAM319:10	*P. miliaceum*	Grain with husk	531–640 AD	Structure of tomb and unearthed cultural relics	A small bag
64TAM10:6	*T. aestivum*	Straw and chaff	617–661AD	Unearthed nine documents with dating texts from 617 AD to 661 AD	Stored in a earthen pot
64TAM10:6	*Panicum miliaceum*	Leaf sheath	ibid	ibid	ibid
64TAM13:15	Porridge made from *P. miliaceum*		During the Tang Dynasty (618–907 AD)	Structure of tomb and unearthed cultural relics	A lump
73TAM532:1	*P. miliaceum and S. italica*	Grain with husk	During the Tang Dynasty (618–907 AD)	Structure of tomb	A small bag
73TAM519:18	*T. aestivum*	Straw and chaff	640–642AD	Unearthed document with a dating text 640 AD and epitaph dated 642 AD	Used as filling of pillow
73TAM519:18	*H. vulgare* var. *coeleste*	Chaff	ibid	ibid	ibid
72TAM209:64	*S. italica*	Grain with husk	658AD	Unearthed epitaph dated 658 AD	Unclear in the original record
72TAM209:66	*T. aestivum*, *H. vulgare* var. *coeleste*	Grain	ibid	ibid	ibid
72TAM209:68	*Cannabis sativa*	Achene	ibid	ibid	ibid
64TAM5:37	*S. italica*	Grain with husk	665–668 AD	19 unearthed documents with dating texts from 665 AD to 668 AD	A small bag
64TAM37:11	Cakes made from *T. aestivum*		768 AD	Unearthed document with a dating text 768 AD	5

### Macrofossil analysis

Length, width and thickness of the cereals were measured with a vernier caliper. The mean values, ranges and standard deviations for each sample population were also calculated (Table 2). All the macroremains were observed and photographed under a stereomicroscope. Samples of *Oryza sativa* were also examined and photographed using a JSM-6610LA scanning electron microscope (SEM) at an accelerated voltage of 20 kV to observe the more detailed morphology of the samples' epidermal cells.

**Table 2:** Statistical results of macrobotanical samples.

Sample	Length (mm)	Range of length (mm)	Width (mm)	Range of width (mm)	Thickness(mm)	Range of thickness(mm)	Count number
Common millet with palea and lemma	3.02±0.09	2.83–3.22	2.19±0.16	1.65–2.39	1.75±0.11	1.37–1.92	50
Caryopsis of common millet	2.38±0.04	2.31–2.43	2.06±0.06	1.98–2.18	1.49±0.08	1.40–1.68	10
Foxtail millet with palea and lemma	2.19±0.06	2.05–2.29	1.78±0.06	1.60–1.89	1.50±0.06	1.39–1.66	50
Caryopsis of foxtail millet	1.67±0.03	1.61–1.72	1.59±0.03	1.54–1.64	1.31±0.10	1.18–1.45	10
Wheat	5.87±0.49	4.90–6.51	2.74±0.21	2.39–3.15	2.60±0.18	2.24–2.85	16
Barley	7.53		3.15		2.72		1
Naked barley	6.03±0.57	5.09–6.97	2.97±0.29	2.49–3.50	2.17±0.25	1.69–2.72	50
Cannabis	4.23±0.44	3.27–4.96	3.30±0.32	2.61–3.76			18
Rice	7.52±0.45	7.12–8.01	3.38±0.16	3.25–3.56	1.54±0.10	1.47–1.65	3

### Microfossil analysis

To determine which plant taxa used during the preparation of the ancient dumplings, cakes, and porridge, impossible to identify with the naked eyes, the samples' starch grains, phytoliths, and bran fragments were extracted and then analyzed under a microscope. In order to identify the ancient starch grains, phytoliths, and bran fragments, one-to-one comparisons with modern samples from economic plants, especially cereals, native to the study region and the published cross-references were performed [17]–[22].

The extraction of starch grains and phytoliths followed well-established methods from published references, *e.g.* [14]. One hundred grains of each sample were measured to obtain data on the length of the starch grains. Previous work has demonstrated that starch grains below 5 µm in size are rarely used in the diagnosis of taxa; therefore, only those exceeding 5 µm in size were counted and calculated [23].

Extraction of bran fragments was conducted. First, about 1.5 cm^3^ of materials were first scraped into a 20 ml beaker. Next, five ml of nitric acid were added to the beaker which was then placed in an electrically heated thermostatic for 10 minutes. During the heating process, the beaker contents were occasionally stirred from which the liquid was then removed and put into a 15 ml labeled snap-cap centrifuge tube. Deionized water was added to the tube which was subsequently centrifuged at 3000 rpm for 5 minutes. The supernatant was then discarded without disturbing the residues at the bottom of the centrifuge tube. Deionized water was again added to the tube. These 3 steps were repeated 3 times. Finally, most of the supernatant was discarded, but allowed to dry at room temperature. An aliquot of the centrifuged residue was then placed on a glass slide in glycerin, mixed with a plastic rod, to which a cover slip was affixed. Slides were then observed and photographed under a light microscope.

## Results

### Macrofossil analysis

**Figure 3:**
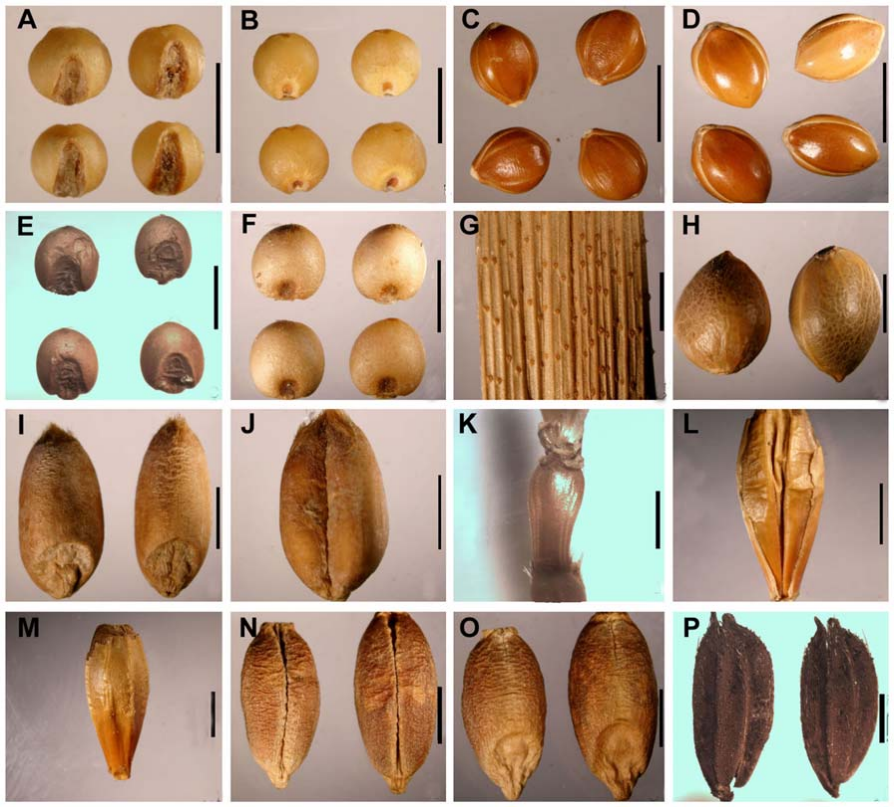
Materials chosen for macrofossil analysis. (A), (B) caryopses of *Setaria italica*; (C) grains with palea and lemma of *Setaria italica*; (D) grains with palea and lemma of *Panicum miliaceum*; (E), (F) caryopses of *Panicum miliaceum*; (G) a leaf sheath of *Panicum miliaceum*; (H) fruits of *Cannabis sativa*; (I), (J) caryopses of *Triticum aestivum*; (K) rachis segment of *Triticum aestivum*; (L), (M) one grain with palea and lemma of *Hordeum vulgare*; (N), (O) caryopses of *Hordeum vulgare* var. *coeleste*; (P) grains with palea and lemma of *Oryza sativa*. (Scale bar: 2 mm).

In the present study, seven cereal plant species were identified from the Astana Cemeteries, including *Setaria italica*, *Panicum miliaceum*, *Cannabis sativa*, *Triticum aestivum*, *Hordeum vulgare*, *Hordeum vulgare* var. *coeleste*, and *Oryza sativa* (Fig. 3). Among them, only one grain of *Hordeum vulgare* with husk was discovered. In view of the fact that plants of *Hordeum vulgare* and *H. vulgare* var. *coeleste* share similar growth conditions, it is probable that a few plants of barley grew together with those of *Triticum aestivum* and/or *Hordeum vulgare* var. *coeleste* in the same field.

### Starch grain analysis

**Figure 4:**
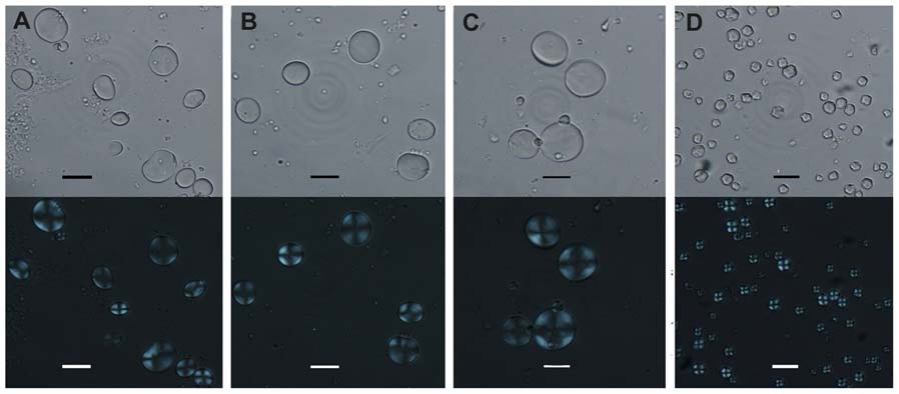
Starch grains from modern plants. The upper row photographs showing starch grains under transmitted light; and the lower row showing corresponding ones under polarized light. (A) starch grains from wheat (*Triticum aestivum*); (B) starch grains from barley (*Hordeum vulgare*); (C) starch grains from naked barley (*Hordeum vulgare* var. *coeleste*); (D) starch grains from modern common millet (*Panicum miliaceum*). (Scale bar: 20 µm).

**Figure 5:**
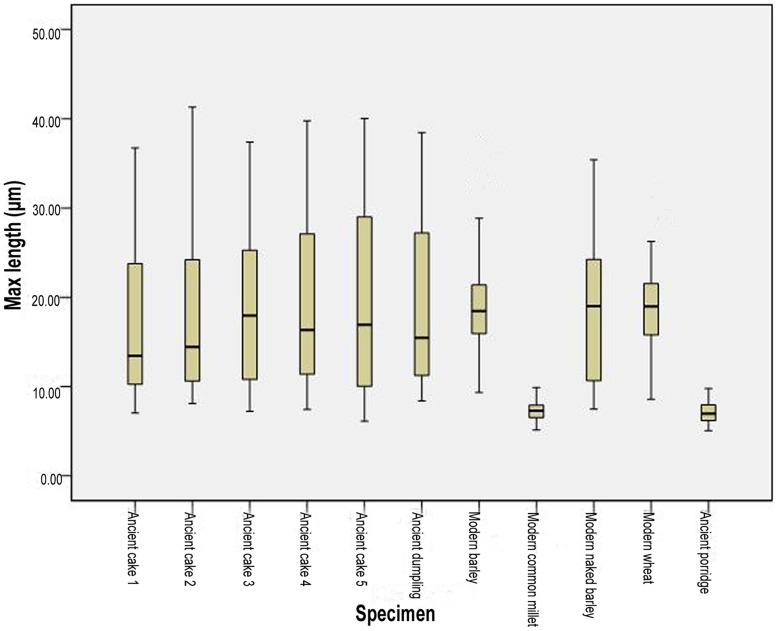
Comparison of starch size ranges (maximum length) between archaeological samples and modern references.

**Figure 6:**
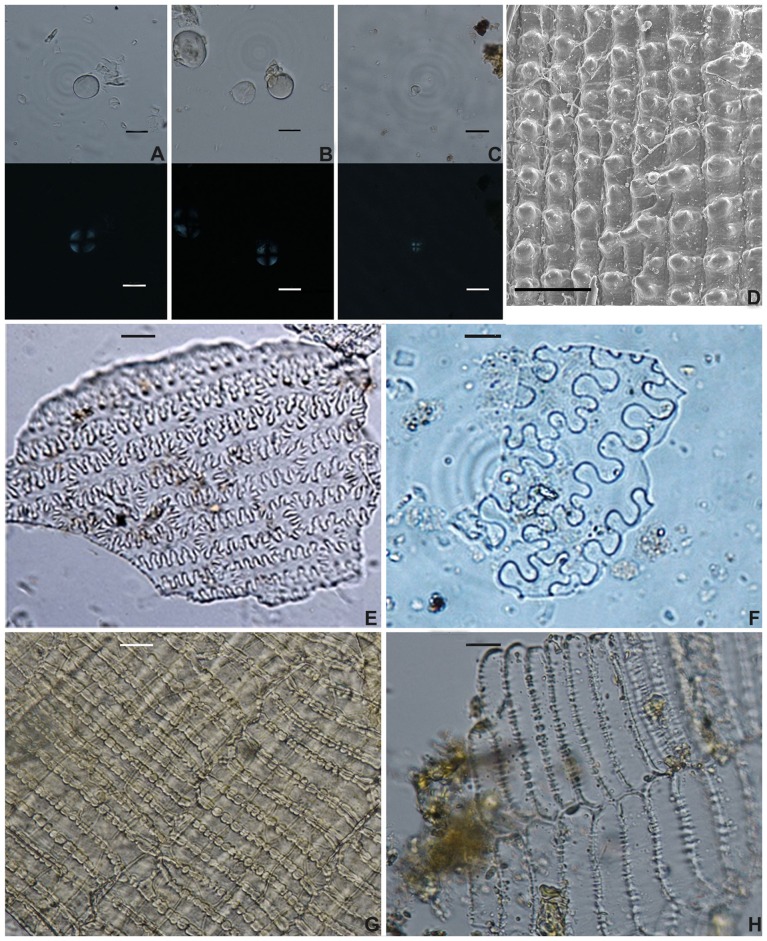
Microfossils extracted from ancient processed food. (A) starch grains from dumpling. Scale bar = 20 µm; (B) starch grains from cakes. Scale bar = 20 µm; (C) starch grains from porridge. Scale bar = 20 µm; (D) silicified epidermis from rice glume. Scale bar = 200 µm; (E) phytoliths from porridge. Scale bar = 20 µm; (F), (G) phytoliths from dumpling. Scale bar = 20 µm; (H) bran fragments from dumpling. Scale bar = 20 µm; (I) bran fragments from cakes. Scale bar = 20 µm.

The starch grains extracted could be divided into two distinctive types. Type 1 were large simple grains (10–40 µm) (Table 3) (Figs. 4, 5), circular/oval in outline, with centric and faint hilum. When rotated into side view, they assumed a lenticular form. Some had demonstrable lamellae (Fig. 6A, B). All cakes and dumplings possessed this type of starch grain. According to modern reference collections and published references [17], [21], [24], [25] (Fig. 4A–C), this type of starch grain could be interpreted as one of the crops belonging to Triticeae. It is most likely that they were derived from *Triticum aestivum*, *Hordeum vulgare*, or *H. vulgare* var. *coeleste*. Type 2 was smaller (4–10 µm) (Table 3) (Figs. 4, 5), sub-rounded and polygonal overall in shape, with a central and faint hilum, and occasional fissures, with no lamellae. Most of the extinction crosses were bilaterally symmetrical. Starch grains extracted from the ancient porridge fell into this type, which could be identified as belonging to *Panicum miliaceum* (Fig. 6C) (compared with Fig. 4D, [22], [26])

**Table 3:** Starch grain size of modern and archaeological samples.

Material	Maximum length (µm)	Range of maximum length (µm)	Count number
Modern wheat	18.85±4.53	8.59–30.74	100
Modern barley	18.65±4.51	9.33–35.42	100
Modern naked barley	18.48±7.66	7.48–35.41	100
Ancient dumpling	19.17±8.80	8.40–38.46	100
Ancient cake 1	17.22±8.29	7.03–36.73	100
Ancient cake 2	18.02±8.98	8.12–41.29	100
Ancient cake 3	18.44±8.28	7.21–37.38	100
Ancient cake 4	19.29±9.26	7.43–39.78	100
Ancient cake 5	19.61±10.29	6.11–40.03	100
Modern common millet	7.29±1.03	5.15–9.90	100
Ancient porridge	7.08±1.16	5.06–9.77	100

### Phytolith analysis

All husk phytoliths extracted from porridge had smooth surfaces without any papillae; the epidermal long cell walls were η-undulated. Moreover, the ending structures of the epidermal long cells were of the cross-finger type (Fig. 6E). In addition, the average R value (ratio of the width of endings interdigitation to the amplitude of undulations) was 0.82, which is in accordance with that of *Panicum miliaceum* (0.79±0.12) [20] (Table 4). In this case the husk phytoliths extracted from the porridge belonged to *Panicum miliaceum*. This scenario agrees well with the results of the starch grain analysis. However, the phytolith morphology of the ancient dumpling revealed that the epidermal long cell walls were Ω-undulated and the ending structures of epidermal long cells were of the cross-wavy type (Fig. 6F). Fifty phytoliths extracted from the dumpling were also measured and statistically analyzed and found to have an average R value of 0.38, which fit quite well with that of *S. italica* (0.33±0.11) [20] (Table 4). The phytoliths from the dumpling were consequently identified to be from *S. italica*.

**Table 4:** Measured data of dendriform epidermal long cells and the R value of samples.

Material	W (µm)	(H1+H2)/2 (µm)	R value	Count number
Dumpling	4.72±1.04	13.28±3.90	0.38±0.10	50
Porridge	10.78±1.71	13.15±1.72	0.82±0.10	50

### Bran fragments analysis

As listed in Fig. 6G and H, the extracted transverse cells of bran fragments were of a hexagonal or pentagonal shape. The long side walls of these transverse cells were extremely thick, with attached and exceedingly large pits. In contrast, the end walls were sometimes pitted, but not usually thickened. In addition, each side presented a narrow curvature and exhibited a zig-zag pattern, the most prominent characteristic of *T. aestivum* [19], [27]. Accordingly, the transverse cells from the dumpling and cakes were identified as belonging to *T. aestivum*.

According to these observations, the components of the ancient cakes, porridge, and dumpling became increasingly clear. The bran analysis together with the starch grain analysis showed that the ancient cakes were made from *T. aestivum*. Accordingly, the bran analysis together with the phytolith analysis suggested that the ancient dumping could have been made from the flour of *T. aestivum* and *S. italica*. However, the phytolith analysis combined with starch grain analysis showed that the ancient porridge was most likely constituted of *P. miliaceum*.

## Discussion

To date, a series of agricultural products together with processed food remains have been discovered in Turpan. For instance, plant remains of *Triticum aestivum*, *Panicum miliaceum*, as well as *H. vulgare* var. *coeleste* have been unearthed in the Yanghai tombs of the Gushi Culture (also called Subeixi Culture) [8]. Ancient foodstuffs, including caryopses of *Panicum miliaceum*, together with noodles, cakes, made from the same, have been discovered in the Subeixi site [14]. There have also been cereal remains unearthed in the other sites belonging to this period. As stated in the site description, the main part of the Turpan Basin consists of the Gobi Desert, and its climate is dry and hot. Thus, it is not strange that all the relative sites are located and found on the secondary mesa of an ancient river or wetland, where it was possible to conduct agricultural activities. Due to water limitation and low productive capability, individuals that inhabited this area were only able to grow a few species of cereal over the past approximate 1000 years (3000-2100 BP). Nomadic peoples still led a pastoral life, and cereals occupied only a small part of their diet.

However, during the period of the Western Han dynasty (202 BC-9 AD), the Han people began migrating to Turpan and witnessed an extremely important stage of development. With the further smoothing of the Silk Road and the migration of people from the eastern part of China, the constant collision and exchange of diverse cultures brought a high degree of development and prosperity in many aspects [28]. This is fully reflected in the development of plant cultivation and agricultural techniques. The present study demonstrates that varieties of cereal crops had greatly improved the lifestyle of the ancient Turpan people during this period. Not only *T. aestivum*, *H. vulgare*, *H. vulgare* var. *coeleste*, and *P. miliaceum*, which had previously been unearthed at archaeological sites belonging to the Subeixi culture, but *S. italica*, *O. sativa* and *Cannabis sativa* were also unearthed. To date, this represents the largest collection of *S. italica* ever uncovered in this region. Even *O. sativa* was uncovered among these plant remains. Although there is possibility that these rice remains were imported from other regions, they might also have been cultivated locally, like their modern counterparts. Agricultural techniques also improved during this period. Previous studies have shown that after the period of the Western Han dynasty, various advanced implements of production spread into Turpan, especially iron tools, having a great impact on local agricultural production [29]. In addition, it is known that water resources were very scarce in the extremely dry environment of Turpan. However, according to ancient written records preserved in the Astana Cemeteries, water from adjacent areas, including the Mutougou Valley and the Tuyugou Valley, were efficiently utilized by the local people with an irrigation system well-managed by the local government. As the people that migrated from the eastern part of ancient China brought advanced agricultural techniques together with a modulation by political means, such as tax collection, much progress was made in Turpan during this period. The more advanced agriculture not only improved the quality of lifestyle of the local people, but also helped the development of commerce [15].

**Figure 7:**
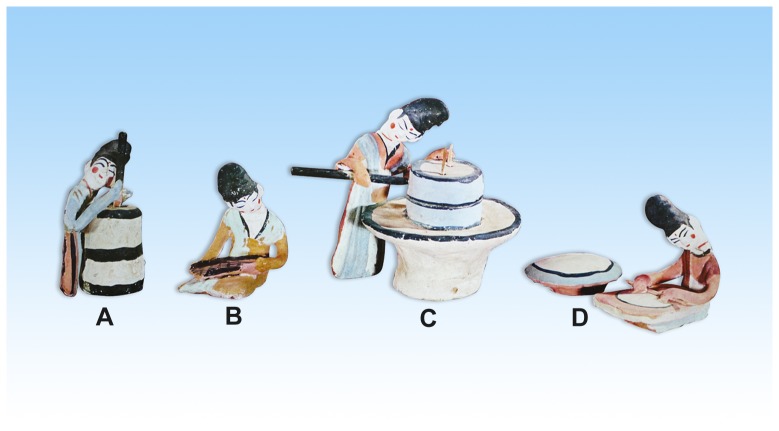
Painted figurines of working women excavated from the Astana Cemteries. They are 9.7–16 cm in height, with vivid expressions and gestures. All of them are deposited in the Xinjiang Uyghur Autonomious Region Museum. (A) dehusking rice with mortar and pestle. (B) winnowing cereal crop. (C) milling flour. (D) baking pan cake (provided by Prof. Yongbing Zhang).

Ancient written records unearthed from the Astana Cemeteries also provide invaluable information about ancient agricultural practices in this region. In accordance with archaeological excavations, written records show that vegetation food played an important role in the daily life of local inhabitants, and that the main cereals of the ancient Astana peoples were *T. aestivum*, *P. miliaceum*, *S. italica*, as well as, *H. vulgare* var. *coeleste*, with the first three more likely important than the latter, which was also consumed as fodder. Furthermore, it is believed that *T. aestivum* flour was processed into fast food for long distance use by travelers, while some fine flour was made into cakes with meat for the consumption of the upper echelons of the society. *T. aestivum* flour was also made into cakes of different styles. Written records also indicate that porridge was usually made from *P. miliaceum* or *S. italica*, with meat sometimes added into the mixture for those of higher status. However, ordinary porridge made from only *P. miliaceum* or *S. italica* was usually consumed by monks, wage earners, as well as poor people [15]. In contrast to the above cereals, fruits of *O. sativa* and *C. sativa* were consumed only in small amounts, also in accordance with archaeological excavations, as only 18 fruits of *C. sativa* as well as a few pieces of *O. sativa* were discovered. In addition, painted figurines were also unearthed from the Astana Cemeteries, including the vivid figurines of working women processing cereal (Fig. 7). In conclusion, such a rich variety of food crops as well as their by-products no doubt greatly improved the living standards of the region's indigenous people and also provided the impetus for a cultural prosperity. In addition, these food remains, particularly the elaborate cakes, reflect that diet was not just for food but also for a higher spiritual enjoyment for certain Turpan ancestors.

As a crossroad of cultures between the East and West, Turpan played an important role in ancient times. An abundance of Persian-style textiles as well as Sassanid silver coins and Roman golden coins have been discovered in the Astana Cemeteries [16]. However, the local food culture was hugely impacted by the eastern region, that is to say, that of the inland areas of ancient China. Until the Tang dynasty (618–907 AD), the manners and customs of the Turpan region were similar to those of Central China. Concurrently, dietary customs of the inland areas also spread there. Large amounts of typical Chinese traditional food remains, especially the dumpling and cakes previously described, have been discovered in the Astana Cemeteries. Observing these ancient dumplings of a thin skin and stuffing, it is surprisingly difficult to distinguish them from their modern counterpart. Written records show that dumplings became popular during the Southern and Northern dynasties (420–589 AD). However, as the components of a dumping are soft and easily perishable, no physical evidence has ever been reported, except for those of the Astana Cemeteries. The Astana Cemeteries contain the earliest physical evidence of dumplings. Even now, dumplings still prevail in most areas of China and the primary cereal ingredient is *T. aestivum*. Compared with its modern counterpart, this study demonstrates that the ancient dumpling unearthed in Turpan was made from a compound flour of *T. aestivum* and *S. italica*. This type of dumpling might have been more suitable to the taste of the ancient Turpan people, but also might indicate an extinct production technology. To resolve this question, more dumplings from the Astana Cemeteries need to be sampled and analyzed.

## Conclusion

Through the study of the food remains and cereal macrofossils unearthed at the Astana Cemeteries, this study provides a fuller insight into the burial ritual, vegetation diet and dietary culture of the Turpan region during the Jin and Tang dynasties (3^rd^ to 9^th^ centuries). This research indicates that with the migration of the Han people to Turpan, the food culture of central China began to play a primary role in the diet of the local people. Furthermore, as an important exchange center and a crucial transport hub on the ancient Silk Road, Turpan's key role in the cultural exchange between East and West became more significant during this period.
